# Revisiting cosmic microwave background radiation using blackbody radiation inversion

**DOI:** 10.1038/s41598-020-80195-3

**Published:** 2021-01-13

**Authors:** Koustav Konar, Kingshuk Bose, R. K. Paul

**Affiliations:** Department of Physics, Birla Institute of Technology, Mesra, Ranchi, Jharkhand 835215 India

**Keywords:** Mathematics and computing, Physics

## Abstract

Blackbody radiation inversion is a mathematical process for the determination of probability distribution of temperature from measured radiated power spectrum. In this paper a simple and stable blackbody radiation inversion is achieved by using an analytical function with three determinable parameters for temperature distribution. This inversion technique is used to invert the blackbody radiation field of the cosmic microwave background, the remnant radiation of the hot big bang, to infer the temperature distribution of the generating medium. The salient features of this distribution are investigated and analysis of this distribution predicts the presence of distortion in the cosmic microwave background spectrum.

## Introduction

A blackbody is an ideal object which can absorb all of the incident radiation of all frequency. The total power radiated per unit frequency per unit solid angle by a unit area of a blackbody emitter can be expressed by Planck’s law^1,2^

1 $${\text{P}}\left( \upnu \right) = \frac{{2{\text{h}}{\upnu ^3}}}{{{{\text{c}}^2}}}\frac{1}{{{{\text{e}}^{\frac{{{\text{h}}\upnu }}{{{\text{kT}}}}}} - 1}}$$

where ν is frequency, T is the absolute temperature, h is Planck’s constant, k is Boltzmann’s constant and c is the speed of light. Usually telescopes are used to measure this power spectrum of any celestial object. But due to its finite field of view a telescope can observe a small portion of the sky at any time. These small portions consist of different blackbody radiators with different temperature T and each of them are in thermal equilibrium. When a collection of blackbodies with probability distribution ${\upalpha }\left( {\text{T}} \right)$ and temperature T is considered, the total radiated power per unit area is given by the integration over the distribution as^3^

2 $${\text{W}}\left( \upnu \right) = \frac{{2{\text{h}}{\upnu ^3}}}{{{{\text{c}}^2}}}\int\limits_0^\infty {\frac{{\upalpha \left( {\text{T}} \right)}}{{{{\text{e}}^{\frac{{{\text{h}}\upnu }}{{{\text{kT}}}}}} - 1}}{\text{dT}}}$$

where W(ν) is the radiated power per unit frequency per unit area and per unit solid angle and ${\upalpha }$(T) is the probability distribution of temperature of the blackbody. The dimension of ${\upalpha }$(T) is $\frac{1}{{\text{K}}}$.

The blackbody radiation inversion problem aims to find the probability distribution of temperature from the radiated power spectrum.

In practice, a set of discrete values of W(ν) are available experimentally. By using this set of data, ${\upalpha }$(T) can be calculated by blackbody inversion method.

For mathematical convenience, a dimensionless parameter G(ν) = $\frac{{c^{2} }}{{2h{\upnu }^{3} }}$ W(ν) is used.

3 $${\text{G}}\left( \upnu \right) = \int\limits_0^\infty {\frac{{\upalpha \left( {\text{T}} \right)}}{{{{\text{e}}^{\frac{{{\text{h}}\upnu }}{{{\text{kT}}}}}} - 1}}{\text{dT}}}$$

Equation (3) is the first kind of Fredholm integral equation and is an ill-posed problem. Bojarski was the first to propose a solution to this problem using Laplace transform with an iterative process^4^. Since then various other methods have been proposed for solving this problem like Tikonov regularization method^5^, universal function set method^6^, Mellin transform method^7^, modified Mobius inverse formula^8^, variational expectation maximization method^9^, maximum entropy method^10^, regularised GMRES method^11^. There are also several other methods available in literature as solutions to this problem^12–15^.

However, the required number of input data is large in the existing method. The number of data points required for successful inversion is 50 in^6^, 50 in^11^ and 32 in^15^. In this paper, a simple and robust method for blackbody radiation inversion is developed which uses 3 input data. The size of programming is also small in comparison to the previous methods. The present method for blackbody radiation inversion reduces the complexity of the overall program significantly.

This method is applied to obtain the probability distribution of temperature of the universe using cosmic microwave background radiation (CMB) from COBE, FIRAS data^16^. “Method and validation” section describes the method and its validation and “Cosmic microwave background radiation” section describes the application of this method in CMB radiation.

## Method and validation

Equation (3) takes all possible values of temperature into consideration; hence the limit runs from zero to infinity. Here it is assumed that the temperature of black body radiators in a collection of blackbodies vary from T_1_ to T_2_ and they have a finite frequency range of ν. Therefore, Eq. (3) can be written as

4 $${\text{G}}(\upnu ) = \mathop \smallint \limits_{{{\text{T}}_{1} }}^{{{\text{T}}_{2} }} \frac{{{\upalpha }\left( {\text{T}} \right)}}{{{\text{e}}^{{\frac{{{{\text{h}\upnu }}}}{{{\text{kT}}}}}} - 1}}{\text{dT}}$$

Using change of variable T = T_1_ + (T_2_ – T_1_)t, Eq. (4) becomes^15^

5 $${\text{G}}(\upnu ) = \left( {{\text{T}}_{{2}} {-}{\text{ T}}_{{1}} } \right)\mathop \int \limits_{0}^{1} \frac{{{\upalpha }\left( {{\text{T}}_{1} + \left( {{\text{T}}_{2} - {\text{T}}_{1} } \right){\text{t}}} \right)}}{{{\text{e}}^{{\left[ {\frac{{{{\text{h}\upnu }}}}{{{\text{k}}\left( {{\text{T}}_{1} + \left( {{\text{T}}_{2} - {\text{T}}_{1} } \right){\text{t}}} \right)}}} \right]}} - 1}}{\text{dt}}$$

6 $${\text{G}}(\upnu ) \, = \mathop \smallint \limits_{0}^{1} {\text{K}}\left( {{\upnu },{\text{t}}} \right){\text{ a}}\left( {\text{t}} \right){\text{dt}}$$

7 $${\text{Where}}\;\;{\text{K}}\left( {{\upnu },{\text{t}}} \right) = \frac{{{\text{T}}_{2} - {\text{T}}_{1} }}{{{\text{e}}^{{\left[ {\frac{{{{\text{h}\upnu }}}}{{{\text{k}}\left( {{\text{T}}_{1} + \left( {{\text{T}}_{2} - {\text{T}}_{1} } \right){\text{t}}} \right)}}} \right]}} - 1}}$$

And a(t) = $${\upalpha }$$(T_1_ + (T_2_ – T_1_) t).

The required interval of a(t) is [0,1].

Equation (6) informs that the problem of solving ${\upalpha }$(T) is equivalent to solving a(t).

In the present article, an analytical function represented by Eq. (8) is proposed as a(t).

8 $${\text{a}}\left( {\text{t}} \right) \, = {\text{k}}_{{1}} {\text{e}}^{{ - {\text{k}}_{2} {\text{t}}^{2} }} \sinh \left( {{\text{k}}_{3}^{2} {\text{t}}} \right)$$

Equation (8) can be expanded as

9 $${\text{a}}\left( {\text{t}} \right) \, = {\text{k}}_{{1}} {\text{e}}^{{ - {\text{k}}_{2} {\text{t}}^{2} }} \left( {\frac{{{\text{e}}^{{{\text{k}}_{3}^{2} {\text{t}}}} - {\text{e}}^{{ - {\text{k}}_{3}^{2} {\text{t}}}} }}{2}} \right)$$

In this method we are trying to obtain the probability distribution of temperature. The nature of the probability distribution is expected to be close to gaussian. So, Eq. (8) is chosen in such a way that for large vales of k_3_, the ${\text{e}}^{{ - {\text{k}}_{3}^{2} {\text{t}}}}$ part in the sine hyperbolic function is very small. When k_2_ $\approx$ ${\text{k}}_{3}^{2}$, Eq. (8) represents a gaussian distribution provided the value of k_1_ is small.

The lower and upper limits of temperatures (T_1_, T_2_) are taken as 1 K and 6 K respectively. The motive behind this choice is that we will use this method to analyse the CMB spectrum and it closely resembles a blackbody radiation at a temperature range similar to T_1_ and T_2_. Then, t = $\frac{{{\text{T}} - 1}}{5}$ or,

10 $${\upalpha }\left( {\text{T}} \right) = {\text{k}}_{{1}} {\text{e}}^{{ - {\text{k}}_{2} \left( {\frac{{{\text{T}} - 1}}{5}} \right)^{2} }} \sinh \left( {{\text{k}}_{3}^{2} \frac{{{\text{T}} - 1}}{5}} \right)$$

where k_1_, k_2_ and k_3_ are three determinable parameters such that the interval of ${\upalpha }\left( {\text{t}} \right)$ is [0,1] and T is absolute temperature.

The data have been simulated by using model function Eq. (11),

11 $${\text{b}}\left( {\text{T}} \right) \, = {\text{e}}^{{ - { }\frac{{\left( {{\text{T}} - {\updelta }} \right)^{2} }}{\gamma }}}$$

Equation (11) is used in Eq. (4) in place of ${\upalpha }\left( {\text{T}} \right)$ and the values of G(ν) are calculated. This process is repeated with different frequencies $\nu$. These simulated data are put in the left-hand side of Eq. (6). Three of such equations for three different values of frequency $\nu$ are obtained. These three equations with three unknowns k_1_, k_2_ and k_3_ are then solved. Thus, the function ${\upalpha }\left( {\text{T}} \right)$ is obtained with these three parameters.

**Figure 1:**
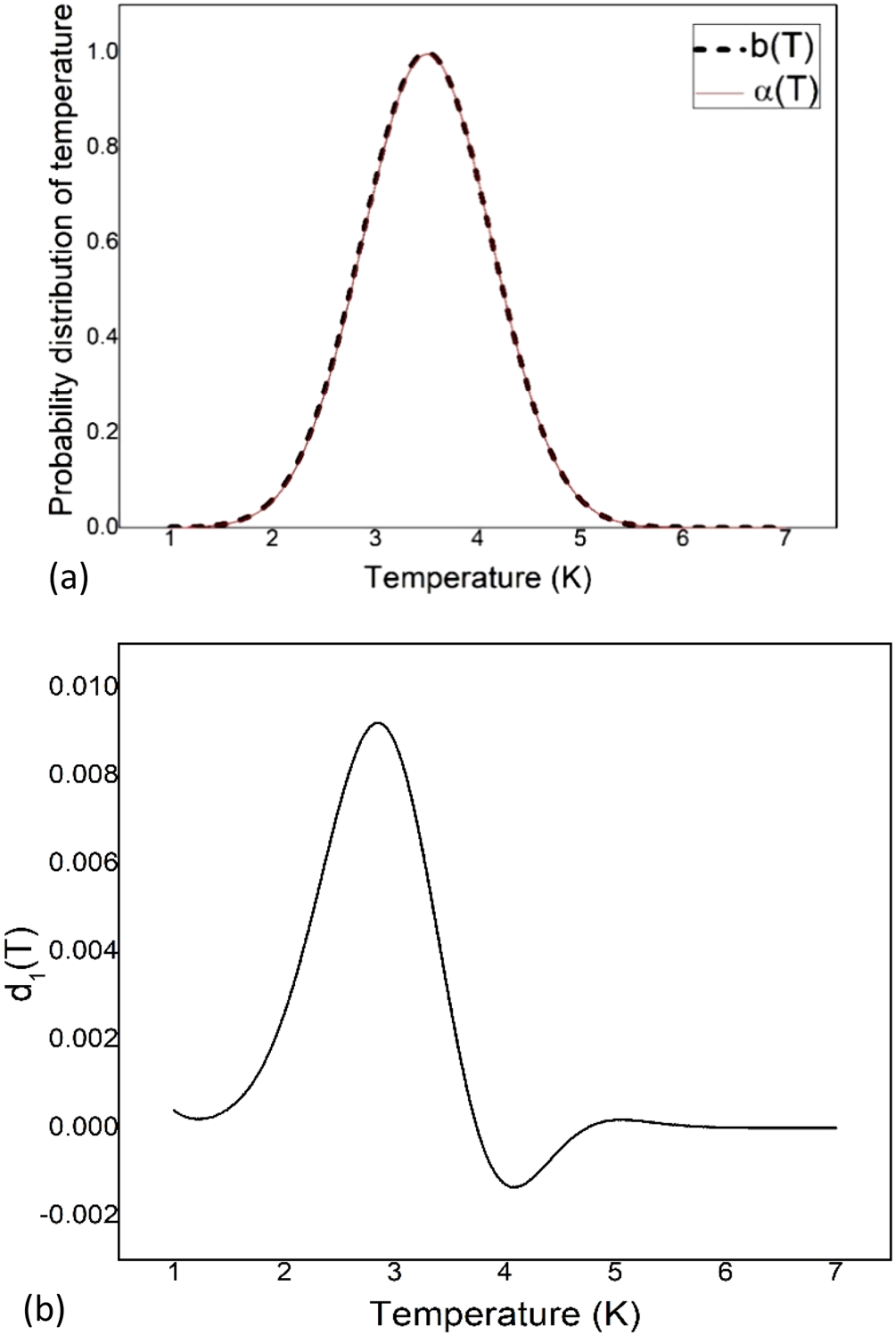
(**a**) Model function b(T) and reconstructed function ${\upalpha }$(T) are plotted against absolute temperature. Here b(T) = ${\text{e}}^{{ - { }\frac{{\left( {{\text{T}} - 3.5} \right)^{2} }}{1}}}$ and three frequencies of 5 $\times$ 10^11^ Hz, 6 $\times$ 10^11^ Hz and 7 $\times$ 10^11^ Hz are used to calculate ${\upalpha }$(T). (**b**) The difference between b(T) and ${\upalpha }\left( {\text{T}} \right)$, d_1_(T) = b(T) − ${\upalpha }\left( {\text{T}} \right)$ is plotted against absolute temperature.

Taking b(T) = ${\text{e}}^{{ - { }\frac{{\left( {{\text{T}} - 3.5} \right)^{2} }}{1}}}$, we calculate ${\upalpha }\left( {\text{T}} \right)$ for three different frequencies of 5 $\times$ 10^11^ Hz, 6 $\times$ 10^11^ Hz and 7 $\times$ 10^11^ Hz. Since we will be using this method in the CMB spectrum, the range of frequency is chosen such a way that it resembles the frequencies in the data we have^16^. It is observed in Fig. 1a that we have reconstructed the temperature distribution that resembles the model temperature distribution. The difference between b(T) and ${\upalpha }\left( {\text{T}} \right)$ is expressed as d_1_(T) = b(T) − ${\upalpha }\left( {\text{T}} \right)$ and it is plotted in Fig. 1b against absolute temperature.

The $\frac{{\Delta {\text{I}}}}{{\text{I}}}$ value obtained from Fig. 1b is 0.0119 for T = 3 K. Here I is the value of b(T) and $\Delta {\text{I}}$ is the value of d_1_(T) for a specific temperature T. The method is sensitive to the chosen frequency. To quantify this sensitivity, we choose sets of frequencies as i, where i includes three frequencies with ${\upnu }_{1}$ = i $\times$ 10^11^ Hz, ${\upnu }_{2}$ = (i + 1)$\times$ 10^11^ Hz and ${\upnu }_{3}$ = (i + 2)$\times$ 10^11^ Hz. This set is then used in Eq. (5) to calculate ${\upalpha }$(T). The standard deviation from the model function is calculated by Eq. (12)

12 $$\sigma \, = \sqrt {\frac{{\mathop \sum \nolimits_{{{\text{j}} = 1}}^{{\text{N}}} \left[ {{\text{b}}_{{\text{j}}} \left( {\text{T}} \right) - {\upalpha }_{{\text{j}}} \left( {\text{T}} \right)} \right]^{2} }}{{\text{N}}}}$$

where N is the number of data used for the calculation of standard deviation and we have taken N = 51.

**Figure 2:**
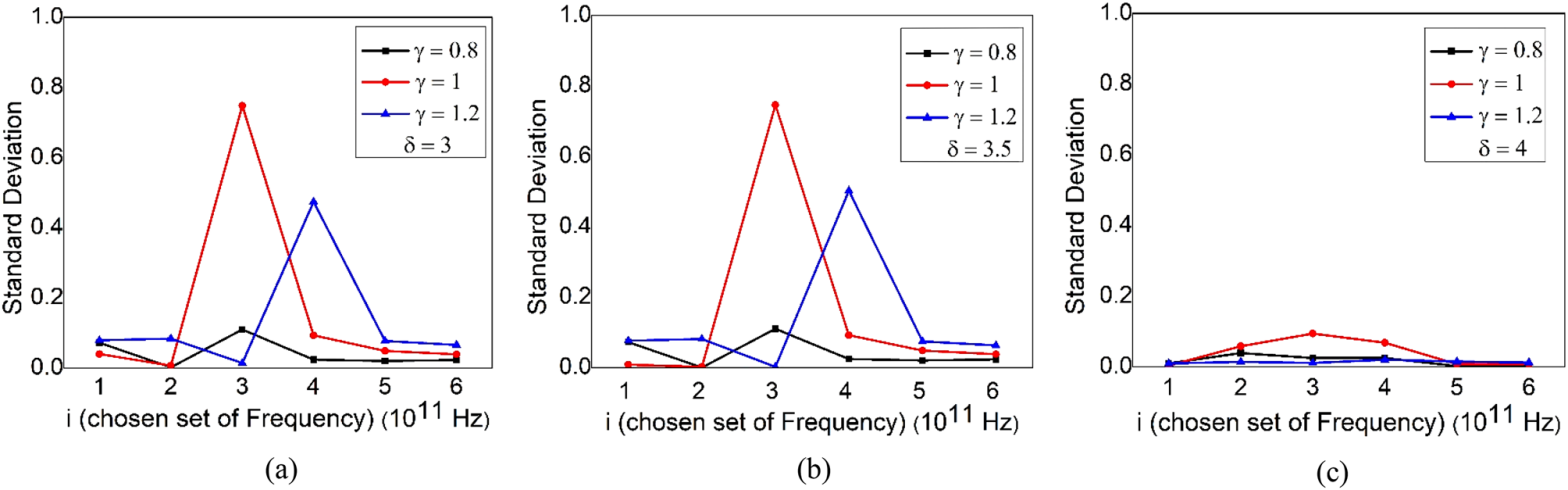
The standard deviation is plotted against the chosen set of frequencies. We have used different profiles of b(T) by varying ${\updelta }$ and $\gamma$ in Eq. (10). For (**a**) ${\updelta }$ = 3, (**b**) ${\updelta }$ = 3.5 and (**c**) ${\updelta }$ = 4. For different $\gamma$ values, the standard deviation differs slightly.

It is inferred from Fig. 2 that the standard deviation is less for the sets of i = 1, 2, 5 and 6. Therefore, it is expected to use either of these set of frequencies. All the values of k_1_, k_2_ and k_3_ that are calculated during the validation, are listed in Table 1.

**Table 1:** It lists all the values of k_1_, k_2_ and k_3_ obtained during the validation process.

Frequency set ($\times$ 10^11^ Hz)	$\gamma$ = 0.8			$\gamma$ = 1.0			$\gamma$ = 1.2		
	k_1_	k_2_	k_3_	k_1_	k_2_	k_3_	k_1_	k_2_	k_3_
**a**									
1,2,3	0.01	33.395	5.162	0.018	29.143	4.859	8.337 × 10^−3^	31.362	5.154
2,3,4	0.278	17.598	3.416	0.623	13.185	2.822	1.86	8.857	1.928
3,4,5	4.019 × 10^−5^	53.165	6.927	2.705 × 10^−4^	42.067	6.219	1.636 × 10^−3^	33.049	5.527
4,5,6	0.012	31.637	5.041	0.039	24.836	4.451	0.072	20.788	4.076
5,6,7	0.014	31.025	4.974	0.042	24.58	4.418	0.071	20.843	4.084
6,7,8	0.015	30.969	4.968	0.025	26.063	4.614	0.068	20.955	4.101
**b**									
1,2,3	0.013	21.065	4.51	3.078 × 10^−3^	26.149	5.107	0.015	19.573	4.418
2,3,4	8.33 × 10^−14^	104.96	10.70	2.466 × 10^−6^	49.555	7.24	0.017	19.225	4.372
3,4,5	0.023	21.621	4.436	8.552	6.04	0.93	9.337 × 10^−8^	52.753	7.752
4,5,6	1.009 × 10^−3^	30.631	5.522	0.025	18.011	4.169	0.498	11.536	2.941
5,6,7	6.54 × 10^−4^	31.802	5.652	4.96 × 10^−3^	24.391	4.92	0.013	20.501	4.515
6,7,8	8.707 × 10^−4^	31.073	5.569	3.306 × 10^−3^	25.358	5.048	9.973 × 10^−3^	21.035	4.595
**c**									
1,2,3	6.062 × 10^−4^	22.673	5.183	6.328 × 10^−5^	28.607	5.876	5.495 × 10^−4^	22.595	5.223
2,3,4	9.062 × 10^−3^	15.737	4.24	0.017	14.432	4.041	1.147 × 10^−3^	20.773	4.991
3,4,5	3.599 × 10^−3^	18.542	4.618	0.041	12.534	3.693	6.771 × 10^−4^	21.983	5.151
4,5,6	5.763	5.339	1.172	0.017	16.119	4.189	2.119 × 10^−3^	19.521	4.809
5,6,7	1.714 × 10^−5^	32.129	6.223	2.976 × 10^−4^	24.619	5.428	1.371 × 10^−3^	20.41	4.938
6,7,8	3.676 × 10^−5^	30.556	6.042	2.417 × 10^−4^	25.033	5.482	9.158 × 10^−4^	21.192	5.052

In this table (a) ${\updelta }$ = 3, (b) ${\updelta }$ = 3.5 and in (c) ${\updelta }$ = 4.0 and the value of $\gamma$ is varied as 0.8, 1 and 1.2 for each ${\updelta }$

## Cosmic microwave background radiation

Cosmic microwave background radiation is the afterglow as predicted by the hot big bang model. The presence of such radiation in the universe was first suggested in the late 1940s^17^. It was only in 1965 when a signal was first detected which was reported to be coming from every direction of the observed sky^18^. This was the first detection of the radiation which later became to be known as cosmic microwave background radiation. The study of CMB can unravel the mysteries of the initial stage of the universe and its evolution for the last 13.7 billion years. Right after its first detection a lot of work has been done on CMB^19–25^.

The first detection showed the radiation to be isotropic, i.e. similar in every direction. But subsequent studies showed that the radiation is in fact anisotropic in nature^26,27^. More recent studies focus on the different types of distortions in the CMB spectrum^28–32^. It suggests that the radiation is not of a blackbody with single temperature, rather it is a superposition of different blackbodies that are at different temperatures. When several blackbodies of different temperatures are mixed together it creates y and ${\upmu }$ type distortions^32,33^. In this paper we have calculated the distortions present in the CMB spectrum.

The blackbody radiation inversion (BRI), as discussed in “Method and validation” section, is applied to the cosmic microwave background radiation for obtaining the temperature of the universe and the probability distribution of temperature. We have used the data of COBE FIRAS to calculate intensity^16^. For the input of the BRI, the spectral irradiance I(λ) is transformed to the power spectrum W(ν) according to the relation

13 $${\text{W}}\left( \upnu \right){\text{d}}\upnu \, = - {\text{ I}}\left( \uplambda \right){\text{d}}\uplambda$$

where λ (= $\frac{c}{{\upnu }}$) is the wavelength. For each value of W(ν) corresponding to a particular frequency ν, we have an integral equation in Eq. (6). Three of such equations are taken to calculate k_1_, k_2_ and k_3_ of Eq. (10). Table 2 shows the values of k_1_, k_2_ and k_3_ that we have calculated.

**Table 2:** k_1_, k_2_ and k_3_ values for different probability distribution function are listed.

	Wavelength in µm	k_1_	k_2_	k_3_
a_1_(T)	1049, 1224, 1468	2.299 × 10^−16^	327.813	14.884
a_2_(T)	918, 1224, 1468	2.811 × 10^−16^	331.555	14.909
a_3_(T)	918, 2451, 1468	2.136 × 10^−16^	331.808	14.938
a_4_(T)	1049, 2451, 1468	2.290 × 10^−16^	324.566	14.846

Three wavelengths are used to obtain three integral equations. The probability distributions are denoted by a_1_(T), a_2_(T), a_3_(T) and a_4_(T) corresponding to a set of frequency.

We have taken the average of these probability distribution functions as M(T),

14 $${\text{M}}\left( {\text{T}} \right) = \frac{{{{\text{a}}_1}\left( {\text{T}} \right) + {{\text{a}}_2}\left( {\text{T}} \right) + {{\text{a}}_3}\left( {\text{T}} \right) + {{\text{a}}_4}\left( {\text{T}} \right)}}{4}$$

Since M(T) is a probability distribution, it should be normalised for the temperature range T_1_ = 1 K to T_2_ = 6 K. We normalise M(T) with normalisation constant 0.982 ($\frac{1}{{\mathop \smallint \nolimits_{1}^{6} {\text{M}}\left( {\text{T}} \right){\text{dT}}}}$ = 0.982). ${\upalpha }$(T) is the normalised probability distribution of temperature.

15 $${\upalpha }\left( {\text{T}} \right) = 0.982 \times {\text{M}}\left( {\text{T}} \right)$$

The moments of different order of ${\upalpha }$(T) are calculated by using Eq. (16)

16 $${\text{nth Moment}} = \mathop \smallint \limits_{1}^{6} \left( {{\text{T}} - {\upmu }} \right)^{{\text{n}}} {\upalpha }\left( {\text{T}} \right){\text{dT}}$$

where n is the order of the moment and µ is the mean value.

First order moment or mean value is calculated as

17 $$\upmu = \int \limits_{1}^{6} {\text{T}} \times {\upalpha }\left( {\text{T}} \right){\text{dT}} \cong 2.69$$

The mean temperature is 2.69 K, which is close to the average value 2.725 K^23^.

Second order moment or variance is calculated as

18 $${\upsigma }^{2} = \mathop \smallint \limits_{1}^{6} \left( {{\text{T}} - {\upmu }} \right)^{2} {\upalpha }\left( {\text{T}} \right){\text{dT = }}0.0{38}$$

So, standard deviation

19 $${\upsigma } = { }\sqrt {{\upsigma }^{2} } = \, 0.{195}$$

σ indicates the uncertainty in temperature which is 0.195 K^23^.

Third order standardised moment or Skewness is calculated as

20 $${{ \upmu }}_{3} = \mathop \int \limits_{1}^{6} \left( {{\text{T}} - {\upmu }} \right)^{3} {\upalpha }\left( {\text{T}} \right){\text{dT}} = { 8}.{3}0{3} \times {1}0^{{ - {4}}}$$

21 $$\upbeta_{{3}} = \frac{{{\upmu }3}}{{{\upsigma }^{3} }} = 1.118 \times 10^{{ - {3}}}$$

An ideal normal distribution has a skewness of 0. We get a positive skewness which describes its deviation from ideal behaviour. A positive skewness suggests that the tail of the distribution right to the mean is more extended than the left-hand side tail^34,35^.

Fourth order standardised moment about mean

22 $${\upmu }_{4} = \mathop \smallint \limits_{1}^{6} \left( {{\text{t}} - {\upmu }} \right)^{4} {\upalpha }\left( {\text{t}} \right){\text{dT}} = { 4}.{429}\times 10^{{ - {3}}}$$

23 $${\text{Excess kurtosis}}\;\;{\upgamma }_{2} = \upbeta_{{2}} - {3}, \quad {\text{ where }}\upbeta_{{2}} = \frac{{{\upmu }^4}}{{{\upsigma }^{4} }} = 3.056.$$

Kurtosis represents the peakedness and tailedness of a distribution. An ideal normal distribution has a kurtosis of 3, so 3 is subtracted from β_2_ to measure the deviation from ideal normal behaviour. In our calculation ${\upgamma }_{2}$ (= β_2_ − 3) yields 0.0563, a positive number. A distribution with positive kurtosis is called Leptokurtic. A positive kurtosis means that the peak of the curve is slightly higher than the normal distribution while the tail and shoulder portion is slightly pushed towards the mean value^34,35^.

From the standard deviation ${\upsigma }$ and mean value µ, a Gaussian function ($\frac{1}{{{\upsigma }\sqrt {2{\uppi }} }}{\text{e}}^{{ - \frac{{\left( {{\text{x}} - {\upmu }} \right)^{2} }}{{2{{\upsigma }}^{2} }}}} { }$) is constructed in Eq. (24).

24 $${\text{s}}\left( {\text{T}} \right) \, = { 2}.0{44} \times {\text{e}}^{{ - \frac{{\left( {{\text{T}} - 2.69} \right)^{2} }}{0.076}}}$$

**Figure 3:**
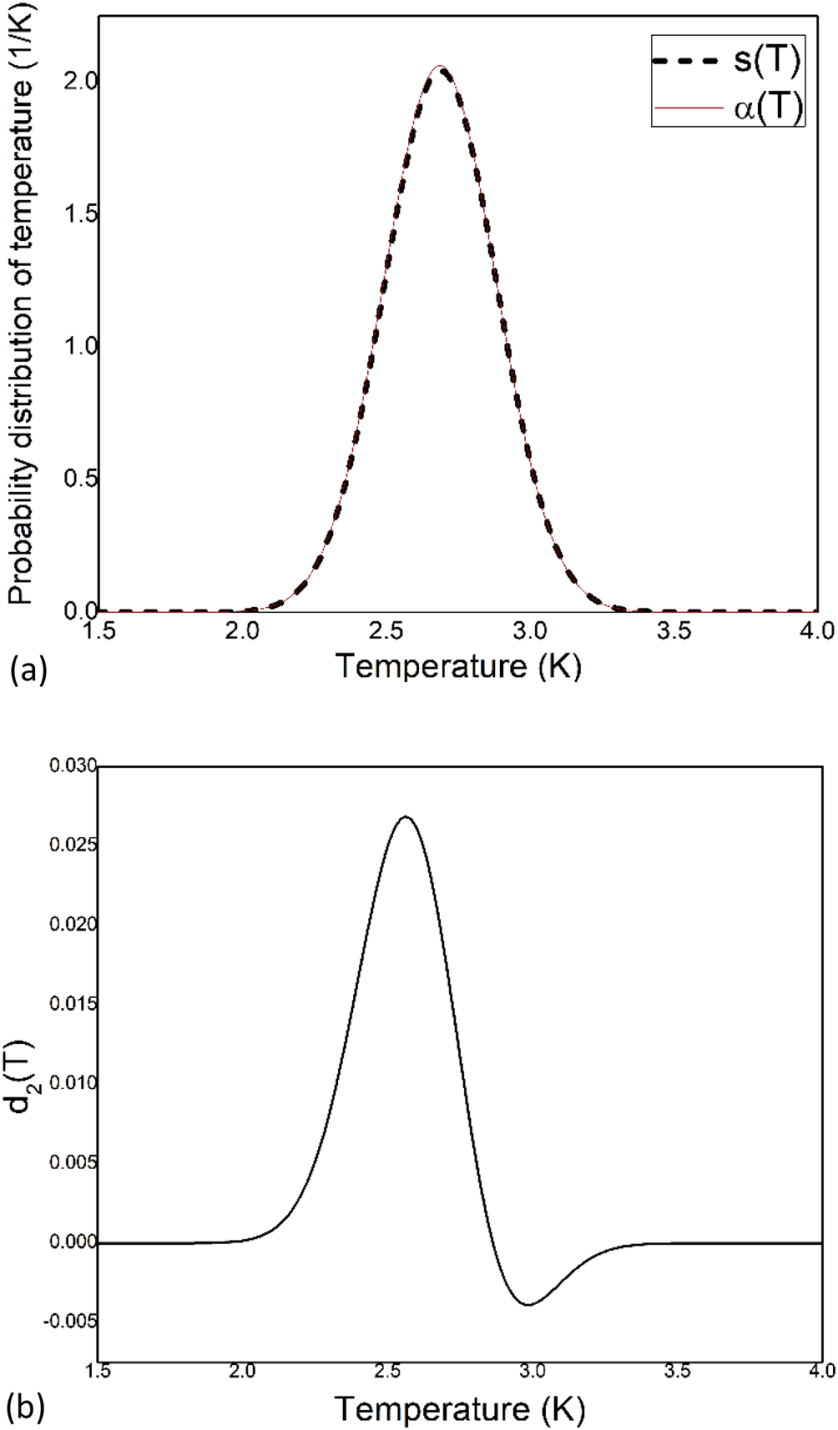
(**a**) The calculated temperature distribution ${\upalpha }$(T) and the Gaussian function s(T) are plotted against absolute temperature. (**b**) The difference between ${{ \upalpha }}$(T) and s(T), expressed as d_2_(T) = ${\upalpha }$(T) – s(T) is plotted against absolute temperature.

${\upalpha }$(T) and s(T) are plotted against absolute temperature in Fig. 3a. And the difference between α(T) and s(T), expressed as d_2_(T) = ${\upalpha }$(T) – s(T) is plotted against absolute temperature in Fig. 3b.

A deviation from the ideal gaussian behaviour is observed. The $\frac{{\Delta {\text{I}}}}{{\text{I}}}$ value obtained from Fig. 3b is 0.0194 for T = 2.5 K. Here I is the value of s(T) and $\Delta {\text{I}}$ is the value of d_2_(T) for a specific temperature T. The deviation in Fig. 3b (0.0194) is larger than the deviation in Fig. 1b (0.0119). So, this deviation in Fig. 3b is not due to the error in the inversion method we have used. A small deviation from ideal Gaussian behaviour is also predicted when non-extensive case is considered^36^. The temperature distribution of CMB is found to be primarily between 2 and 3.5 K.

**Figure 4:**
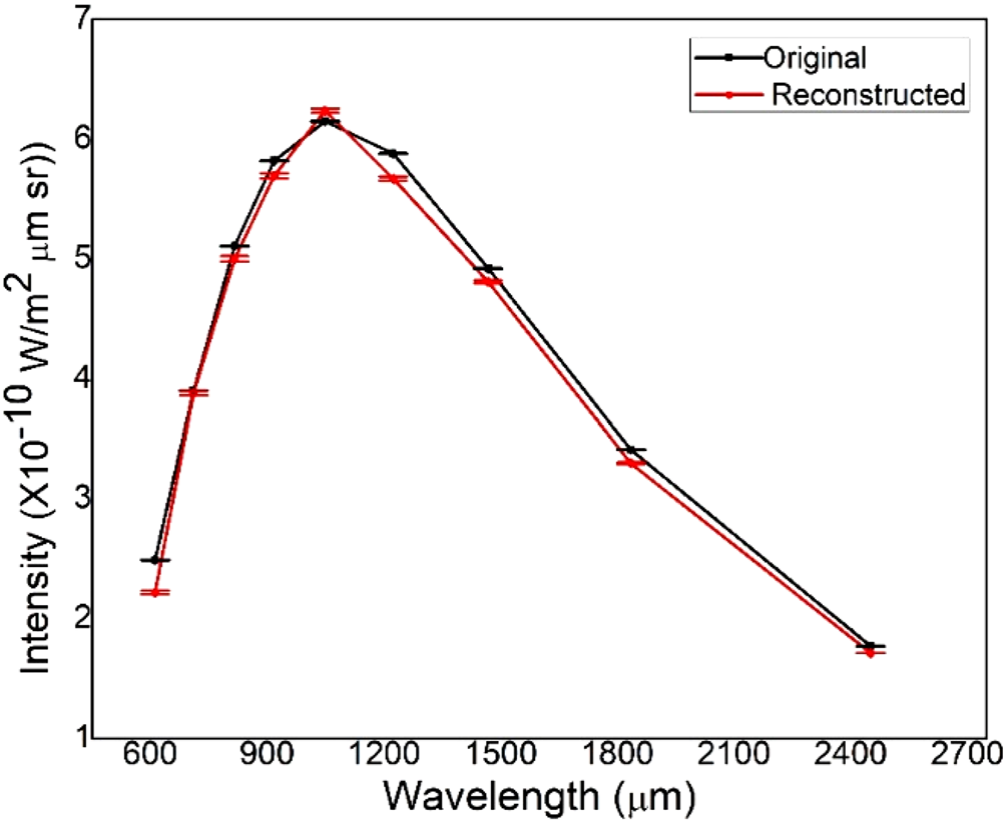
The obtained probability distribution of temperature is used to reconstruct the intensity of the original input data. Both of the intensities are plotted together against wavelength. The error bars are also shown.

To verify the accuracy of our method to obtain probability distribution of temperature, we reconstructed the intensity of the radiation by using the calculated ${\upalpha }$(T) in Eq. (2) for different frequencies ν. W($\nu$) is then converted to I(λ) by using Eq. (13). Figure 4 displays the overlay of reconstructed data on the original data of COBE FIRAS.

The small error bars are not visible in Fig. 4. Hence the values of the intensity and the error are given in Table 3 for the original and reconstructed data. The order of the error in the reconstructed spectrum (~ 10^−13^ W/m^2^ × μm × sr) is larger than the error in the original spectrum (~ 10^−14^ W/m^2^ × μm × sr). It is evident from Fig. 4 that the present method can faithfully reconstruct the original data.

**Table 3:** The values of original and reconstructed data for intensity are listed.

Wavelength (μm)	Original (COBE) (10^−10^ W/m^2^ × μm × sr)	Original uncertainty (COBE) (10^−10^ W/m^2^ × μm × sr)	Reconstructed (10^−10^ W/m^2^ × μm × sr)	Reconstructed uncertainty (10^−10^ W/m^2^ × μm × sr)
612	2.49	1.84 × 10^−3^	2.219	1.3 × 10^−2^
711	3.9	1.187 × 10^−3^	3.88	1.85 × 10^−2^
816	5.11	1.036 × 10^−3^	5.001	2.1 × 10^−2^
918	5.82	7.83 × 10^−4^	5.695	2.1 × 10^−2^
1049	6.15	3.817 × 10^−4^	6.239	1.95 × 10^−2^
1224	5.88	2.022 × 10^−4^	5.67	1.65 × 10^−2^
1468	4.92	1.81 × 10^−4^	4.809	1.15 × 10^−2^
1835	3.41	1.604 × 10^−4^	3.297	6.5 × 10^−3^
2451	1.77	1.099 × 10^−4^	1.713	3 × 10^−3^

The original and reconstructed spectrum uncertainties are also included.

In this paper, the original data of COBE/FIRAS are used as the input in the blackbody radiation inversion problem. These data are thus mathematically processed to obtain the distortion of the CMB spectrum. The standard deviation between the original and reconstructed data is 0.142 $\times$ 10^−10^ W/m^2^ × μm × sr or 5.209 $\times$ 10^−20^ W/m^2^ $\times \;{\text{Hz}} \times$ sr for the wavelength of 1049 μm. The deviation is the distortion present in the CMB spectrum. The spread in the probability distribution of the temperature (Fig. 3a) suggests that there are multiple blackbodies with different temperatures ($\Delta {\text{T}} = {\upsigma }$). Due to this mixing of blackbodies, the original spectrum becomes distorted. So, the calculated deviation is interpreted as the distortion of the CMB spectrum.

The COBE data shows a spectrum similar to a perfect blackbody^21^. But the possible distortions are limited by the maximum sensitivity of the instrument. It has y distortion of |y|< 1.5 $\times$ 10^−5^ and ${\upmu }$ distortion of |${\upmu }$|< 9.0 $\times$ 10^−5^^21^. In our calculation we have obtained the temperature as T_new_ = T [1 + $\left( {\frac{{\Delta {\text{T}}}}{{\text{T}}}} \right)^{2}$] = 2.704 K for T = 2.69 K and $\Delta {\text{T}} = { }$ 0.195 K. The y and ${\upmu }$ distortions are calculated as y = $\frac{1}{2}\left( {\frac{{\Delta {\text{T}}}}{{\text{T}}}} \right)^{2}$ $\approx$ 10^−3^ and ${\upmu }$ = $2.8 \times \left( {\frac{{\Delta {\text{T}}}}{{\text{T}}}} \right)^{2}$ $\approx$ 10^−2^^30^.

The present set of data, collected by the COBE/FIRAS satellite is not sensitive enough to detect the distortions beyond the 10^−5^ order. More precise datasets are required to study these distortions. The TRIS, used between 1996 to 2000, had the limit of ${\upmu } <$ 6.0 $\times$ 10^−5^^37^. A balloon borne instrument ARCADE (Absolute Radiometer for Cosmology, Astrophysics, and Diffuse Emission) used in 2006, had the upper limit of ${\upmu } <$ 6.0 $\times$ 10^−4^^38^. Two new projects, PIXIE^39^ and PRISM^40^ aim to find the distortions with 10^3^–10^4^ times better sensitivity than COBE/FIRAS. PIXIE has $\Delta$I = 5 $\times$ 10^−26^ W/m^2^srHz and detection of |y|= 1 $\times$ 10^−8^ and |${\upmu }$|= 5.0 $\times$ 10^−8^ is possible. PRISM is better than PIXIE with $\Delta$I = 6 $\times$ 10^−27^ W/m^2^srHz and sensitive to y and ${\upmu }$ distortion of ~ 10^−9^.

The distortions obtained in our calculation are limited by the sensitivity of available measured data. The experiments planned in the future^39,40^ are expected to provide data with better precision that will help in carrying out more precise calculations and lead to a better understanding of CMB.

## Discussion

A novel method of blackbody radiation inversion is studied. This technique is then applied to study cosmic microwave background radiation and some of its most important features. We have described the deviation of the temperature probability distribution from ideal gaussian distribution. The distortion in the spectrum, caused due to mixing of blackbodies are mathematically described as well. Our approach is much simpler than the existing techniques and the computational bulkiness is significantly reduced. While we can obtain the probability distribution of the temperature effectively, the present method is not completely general in nature. The frequency range needs to be selected to minimise the error in the calculation.
